# *Cynoglossus semilaevis* ISG15: A Secreted Cytokine-Like Protein That Stimulates Antiviral Immune Response in a LRGG Motif-Dependent Manner

**DOI:** 10.1371/journal.pone.0044884

**Published:** 2012-09-18

**Authors:** Wei Wang, Min Zhang, Zhi-zhong Xiao, Li Sun

**Affiliations:** 1 Key Laboratory of Experimental Marine Biology, Institute of Oceanology, Chinese Academy of Sciences, Qingdao, China; 2 Graduate University of the Chinese Academy of Sciences, Beijing, China; Whitehead Institute, United States of America

## Abstract

ISG15 is an ubiquitin-like protein that is induced rapidly by interferon stimulation. Like ubiquitin, ISG15 forms covalent conjugates with its target proteins in a process called ISGylation, which in mammals is known to play a role in antiviral immunity. In contrast to mammalian ISG15, the function of teleost ISG15 is unclear. In this study, we identified and analyzed the function of an ISG15 homologue, CsISG15, from tongue sole (*Cynoglossus semilaevis*). CsISG15 is composed of 162 residues and possesses two tandem ubiquitin-like domains and the highly conserved LRGG motif found in all known ISG15. Expression of *CsISG15* occurred in a wide range of tissues and was upregulated in kidney and spleen by viral and bacterial infection. In vitro study with primary head kidney (HK) lymphocytes showed that megalocytivirus infection caused induction of *CsISG15* expression and extracellular release of CsISG15 protein. Purified recombinant CsISG15 (rCsISG15) activated HK macrophages and enhanced the expression of immune genes in HK lymphocytes, both these effects, however, were significantly reduced when the conserved LRGG sequence was mutated to LAAG. Further study showed that the presence of rCsISG15 during megalocytivirus infection of HK lymphocytes reduced intracellular viral load, whereas antibody blocking of CsISG15 enhanced viral infection. Likewise, interference with CsISG15 expression by RNAi promoted viral infection. Taken together, these results indicate that CsISG15, a teleost ISG15, promotes antiviral immune response and that, unlike mammalian ISG15, CsISG15 exerts its immunoregulatory effect in the form of an unconjugated extracellular cytokine. In addition, these results also suggest a role for the LRGG motif other than that in protein conjugation.

## Introduction

Interferons (IFNs) play an important role in the innate immunity against viral infection. IFNs bind to their cognate receptors on the target cells and activate the signal transduction pathways involving Jak kinases and the transcription factors of the STAT family [1], [2], which in turn activate the transcription of hundreds of IFN-stimulated genes (ISGs) [3], [4]. Among the identified ISGs are a group of proteins called ISG15, which are small ubiquitin-like proteins induced rapidly by IFN stimulation [5], [6].

ISG15 was first identified in humans as a ∼15 kDa protein derived from a 165-residue precursor [7]. Subsequently, ISG15 homologues were discovered in diverse vertebrate species including fish. All ISG15 proteins possess two ubiquitin-like (UBL) domains and a highly conserved C-terminal LRGG sequence, the latter being known as the ubiquitin conjugation motif [8]. In mammals, both intracellular and extracellular ISG15 have been detected. Intracellular ISG15 are conjugated, via the LRGG motif, to target proteins through a process called ISGylation, which resembles largely ubiquitination, the process of formation of ubiquitin conjugates. Ubiquitination involves three enzymes, i.e., ubiquitin activating enzyme (E1), ubiquitin conjugating enzyme (E2), and ubiquitin ligase (E3). E1 catalyzes adenylylation of the C-terminal di-glycine sequence in the LRGG motif, which is exposed after proteolytic cleavage, while E2 and E3 catalyze transferring of the ubiquitin moiety to the substrate protein [9]. In most cases, the first ubiquitin molecule is attached to the substrate protein through a linkage formed between the C-terminal glycine residue of ubiquitin and a lysine residue of the substrate protein [10]. Poly-ubiquitination is achieved by successive attachment of new ubiquitin molecules to the conjugated ubiquitins. Similar to ubiquitination, ISGylation begins by adenylylation of the C-terminal di-glycine sequence of the LRGG motif, which is followed by successive transfer of ISG15 from E1, E2 and E3 enzymes to the target protein [11]. Unlike ubiquitination, which is known to function in protein and immune regulation [12], [13], the function and biological significance of ISGylation remain elusive. However, recent evidences suggest an involvement of ISG15, in the form of conjugated protein modifiers, in regulation of IFN signaling and in antiviral immunity [9], [14]. Both ubiquitin and ISG15 have been found to exist extracellularly in unconjugated forms. Extracellular ubiquitins are known to inhibit secretion of tumor necrosis factor (TNF)-α and TNF-α mRNA expression from peripheral blood mononuclear cells in response to endotoxin [15]. Likewise, unconjugated extracellular ISG15, which are released from several types of human and murine cells, are known to possess cytokine-like activity [16]–[20].

ISG15-like sequences from a number of teleost species have been reported, most of which were found to be positively regulated in expression by microbial challenge [21]–[26]. Recent studies showed that the ISG15 homologues of Atlantic cod (*Gadus morhua* L.), Atlantic salmon (*Salmo salar*), and goldfish (*Carassius auratus*) are capable of forming conjugates with cellular proteins [27]–[29], and the ISG15 of red drum (*Sciaenops ocellatus*) exhibits immunoregulatory property [30]. However, except these few cases, the activity and biological function of teleost ISG15 are essentially unknown. In this study, we identified an ISG15 homologue, CsISG15, from tongue sole (*Cynoglossus semilaevis*) and analyzed its potential function. Our results showed that CsISG15 is a secreted cytokine-like protein that, unlike mammalian ISG15, possesses antiviral capacity in the unconjugated form.

## Results

### Characterization of the sequence of CsISG15

**Figure 1:**
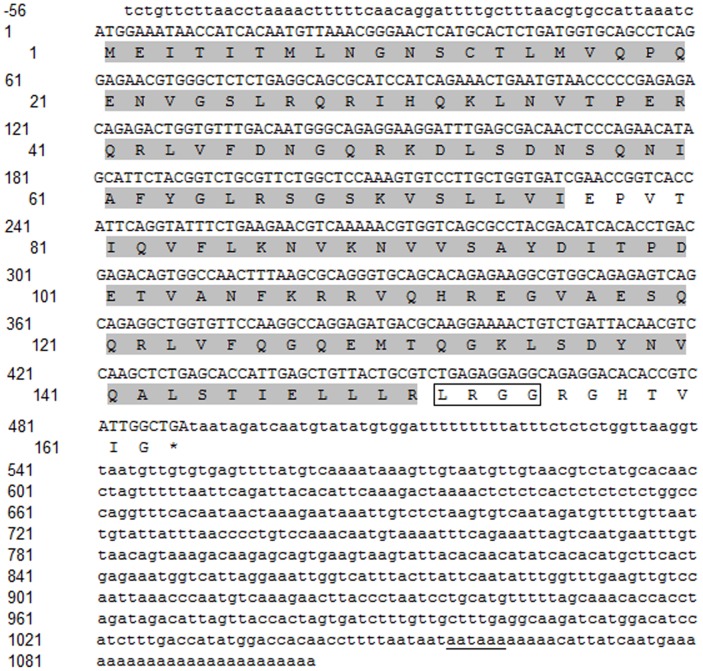
The nucleotide and deduced amino acid sequences of CsISG15. The nucleotides and amino acids are numbered along the left margin. In the nucleotide sequence, the translation start and stop codons are in bold, and the polyadenylation signal is underlined. In the amino acid sequence, the two ubiquitin-like (UBL) domains are shaded, and the ubiquitin conjugation motif (LRGG) is boxed.

The cDNA of CsISG15 is 1159 bp, which contains a 5′-UTR of 57 bp, an open reading frame of 489 bp, and a 3′-UTR of 613 bp (Fig. 1). The cDNA is terminated with a poly-A tail formed by 25 adenines. A putative polyadenylation signal, AATAAA, is located 16 bp upstream the poly-A tail. The deduced amino acid sequence of CsISG15 is 162-residue in length and has a calculated molecular mass of 18.3 kDa and a theoretical pI of 9.7. CsISG15 contains two tandem UBL domains (residues 1 to 76 and 81 to 151, respectively) and the conserved C-terminal LRGG motif, the latter being followed by seven residues that terminate the CsISG15 sequence. No putative signal peptide or glycosylation site was found in CsISG15. Blast analysis showed that CsISG15 shares 41.5–61.6% overall sequence identities with the ISG15 of the teleosts *Oplegnathus fasciatus*, *Epinephelus coioides*, *Paralichthys olivaceus*, *Sebastes schlegelii*, *Anoplopoma fimbria*, *Channa argus*, *Oncorhynchus mykiss*, *Salmo salar*, and *Danio rerio* (Figure S3). The overall sequence identities between CsISG15 and human and mouse ISG15 are 30.5% and 27.1% respectively.

### Tissue specific expression of *CsISG15* under normal physiological conditions and during pathogen infection

**Figure 2:**
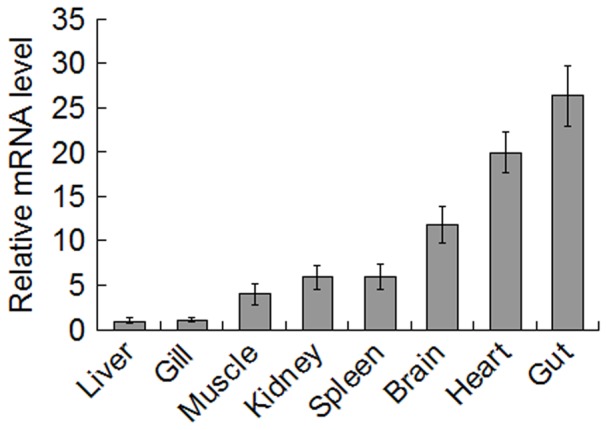
CsISG15 expression in fish tissues. *CsISG15* expression in the liver, gill, muscle, kidney, spleen, brain, heart, and gut of tongue sole was determined by quantitative real time RT-PCR. The expression level in liver was set as 1. Five animals were used at each time point, and for each time point the PCR was performed three times. The results are shown as means±SE (N = 5).

**Figure 3:**
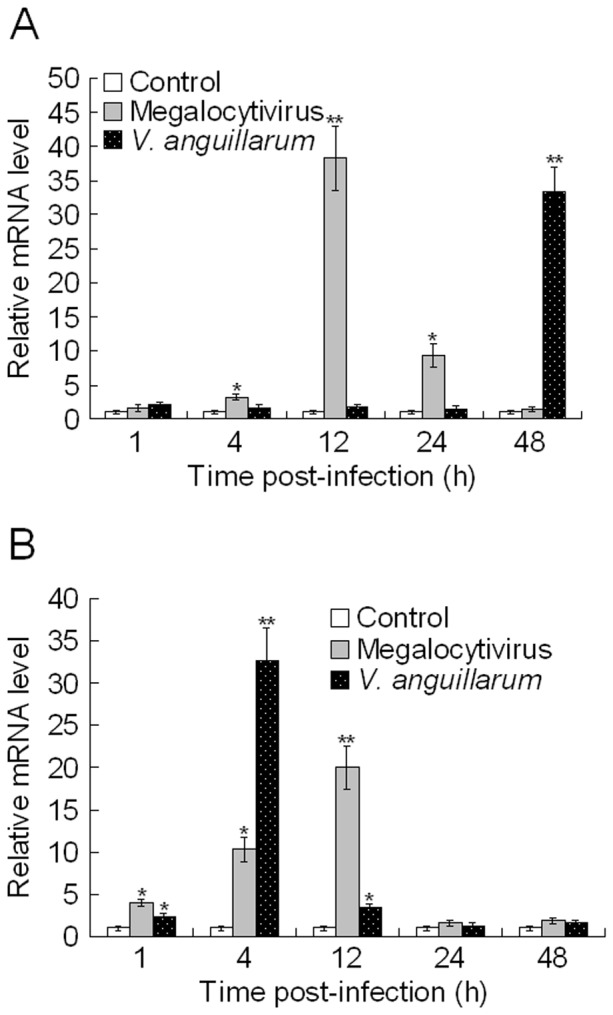
*CsISG15* expression in response to pathogen infection. Tongue sole were infected with megalocytivirus, *Vibrio anguillarum*, or PBS (control), and *CsISG15* expression in kidney (A) and spleen (B) was determined by quantitative real time RT-PCR at various time points. At each time point, the expression level of the control fish was set as 1. Five animals were used at each time point, and for each time point the PCR was performed three times. The results are shown as means±SE (N = 5). Significances between PBS- and pathogen-infected fish are indicated with asterisks. ^**^ *P*<0.01, ^*^ *P*<0.05.

**Figure 4:**
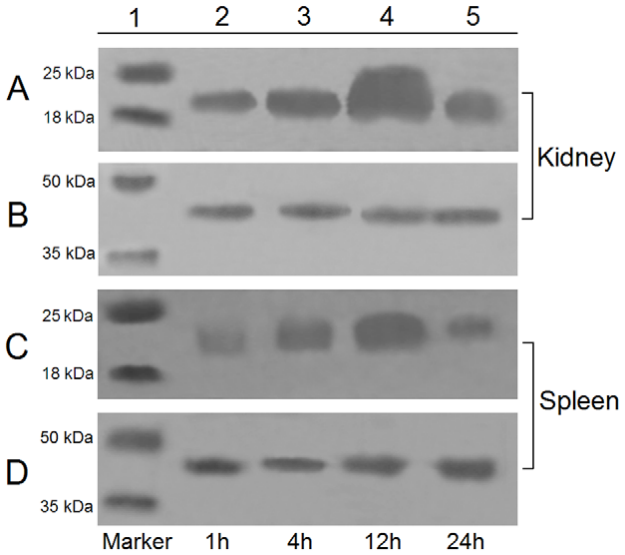
CsISG15 production in kidney and spleen in response to pathogen infection. Tongue sole were infected with megalocytivirus for 1 h, 4 h, 12 h, and 24 h, and CsISG15 production in kidney (A and B) and spleen (C and D) was determined by Western blot with antibodies against rCsISG15 (A and C) or β-actin (B and D).

qRT-PCR analysis showed that under normal physiological conditions, *CsISG15* expression was detected, in increasing order, in liver, gill, muscle, kidney, spleen, brain, heart, and gut (Fig. 2). The expression level in gill, muscle, kidney, spleen, brain, heart, and gut were, respectively, 1.1-, 4-, 5.9-, 6-, 11.9-, 20-, and 26.4-fold higher than that in liver. To examine whether *CsISG15* expression was affected by pathogen infection, tongue sole were infected experimentally with the viral pathogen megalocytivirus or the bacterial pathogen *V. anguillarum*, and *CsISG15* expression in kidney and spleen was subsequently determine by qRT-PCR at 1 h, 4 h, 12 h, 24 h, and 48 h post-infection. The results showed that both megalocytivirus and *V. anguillarum* induced *CsISG15* expression in time dependent manners (Fig. 3). In kidney, megalocytivirus infection caused significant inductions (3.2-, 38.2-, and 9.3-fold respectively) of *CsISG15* expression at 4 h, 12 h, and 24 h post-infection, while *V. anguillarum* infection caused significant induction (33.4 fold) at 48 h post-infection (Fig. 3A). In spleen, both megalocytivirus and *V. anguillarum* induced significant *CsISG15* expression at 1 h, 4 h, and 12 h post-infection, with peak inductions (20- and 32.6-fold respectively) occurring at 12 h and 4 h post-infection respectively (Fig. 3B). Consistent with the qRT-PCR results, Western blot analysis showed that CsISG15 protein increased with megalocytivirus infection and reached peak levels at 12 h in kidney and spleen (Fig. 4).

### CsISG15 expression and subcellular localization in HK lymphocytes

**Figure 5:**
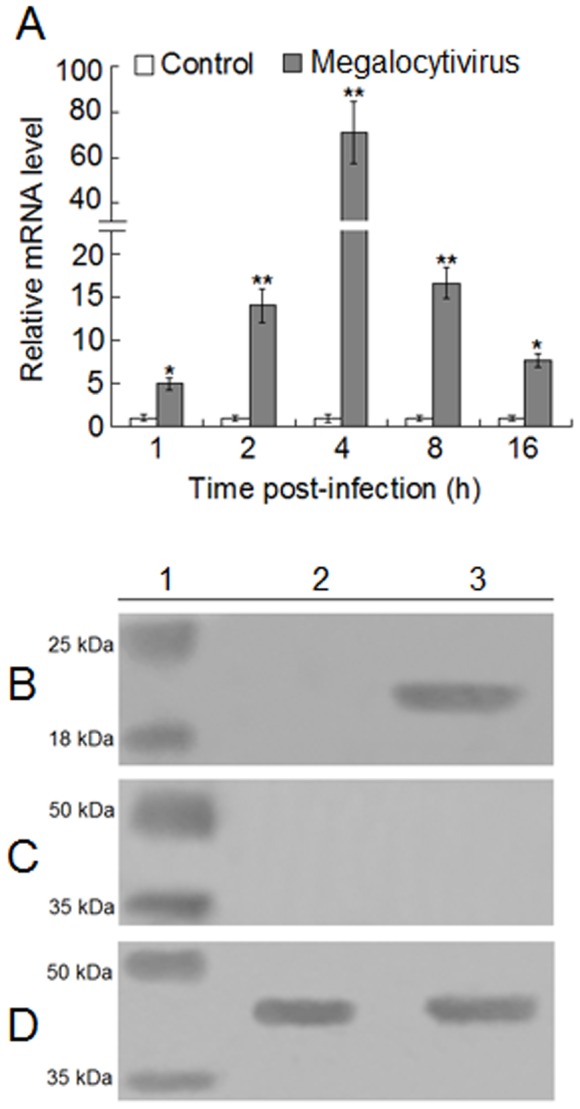
Expression and localization of CsISG15 in lymphocytes. (A) Tongue sole head kidney (HK) lymphocytes were infected with or without (control) megalocytivirus, and *CsISG15* expression was determined by quantitative real time RT-PCR at various time points. At each point, the expression level of the control cells was set as 1. Values are shown as means±SE (N = 3). N, the number of repeated experiments. ^**^ *P*<0.01, ^*^ *P*<0.05. (B, C, and D) Tongue sole HK lymphocytes were infected with (lane 3) or without (lane 2) megalocytivirus for 4 h, and extracellular proteins were subjected to immunoblot with antibodies against CsISG15 (B) or β-actin (C). To demonstrate that the β-actin antibody was able to detect β-actin when it was present, cytoplasmic proteins were prepared and blotted with β-actin antibody (D). Lane 1, protein marker.

To examine the effect of pathogen infection on *CsISG15* expression at cellular level, tongue sole HK lymphocytes were infected with or without megalocytivirus, and *CsISG15* expression was determined by qRT-PCR at 1 h, 2 h, 4 h, 4 h, 8 h, and 16 h post-infection. The results showed that significant inductions of *CsISG15* expression were observed at all the examined time points, with maximum induction (75 fold) occurring at 4 h post-infection (Fig. 5). To examine whether CsISG15 was secreted into the extracellular milieu as it was observed with human ISG15, HK lymphocytes were infected with or without megalocytivirus for 4 h, which, as shown above, was the point when drastic *CsISG15* induction was observed. Extracellular proteins were prepared from the culture supernatant and subjected to immunoblot analysis with antiserum raised against recombinant CsISG15 (Figure S1 and Figure S2) or with antibody against β-actin. The results showed that CsISG15 was detected in the culture supernatant of megalocytivirus-stimulated cells but not in that of unstimulated cells (Fig. 5). Since no β-actin was detected in the protein preparations, it is not likely that the detected CsISG15 was due to cell lysis.

### Biological effect of rCsISG15 and its dependence on the conserved LRGG motif

#### (i) Effect on macrophage activation

**Figure 6:**
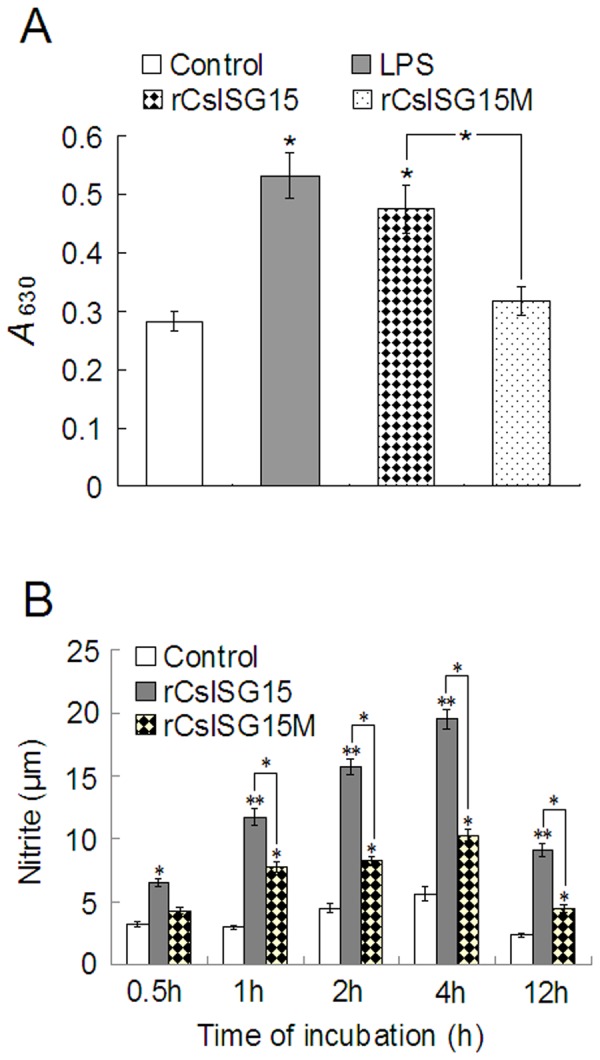
Effect of rCsISG15 and rCsISG15M on macrophage activation. (A) Tongue sole head kidney macrophages were treated with or without (control) rCsISG15, rCsISG15M, or lipopolysaccharide (LPS) for 2 h and examined for respiratory burst. (B) Tongue sole head kidney macrophages were treated with or without (control) rCsISG15 or rCsISG15M for different hours and examined for nitric oxide production. The assays were each performed four times and the results are shown as means±SE. ^**^ *P*<0.01, ^*^ *P*<0.05.

Since, as observed above, CsISG15 was a secreted protein, we examined its potential biological function by determining whether it had any effect on macrophage and lymphocyte immune response. In addition, to examine the functional importance of the LRGG motif, we also prepared and analyzed the effect of a mutant CsISG15, CsISG15M, which bears alanine substitutions at the RG residues of the LRGG sequence (i.e., LRGG was mutated to LAAG). HK macrophages were treated with or without rCsISG15, rCsISG15M, or LPS for various hours and then analyzed for respiratory burst and NO production. The results showed that significantly increased respiratory burst activity was observed in cells treated with rCsISG15 and LPS but not in cells similarly treated with rCsISG15M (Fig. 6A). NO production analysis showed that macrophages treated with rCsISG15M produced significantly higher amounts of NO after 0.5 h, 1 h, 2 h, 4 h, and 12 h treatment, while macrophages treated with rCsISG15M exhibited significant NO induction after 1 h, 2 h, 4 h, and 12 h treatment, however, the induction levels induced by rCsISG15M were significantly lower than those induced by rCsISG15 (Fig. 6B).

#### (ii) Effect on lymphocyte immune response

**Figure 7:**
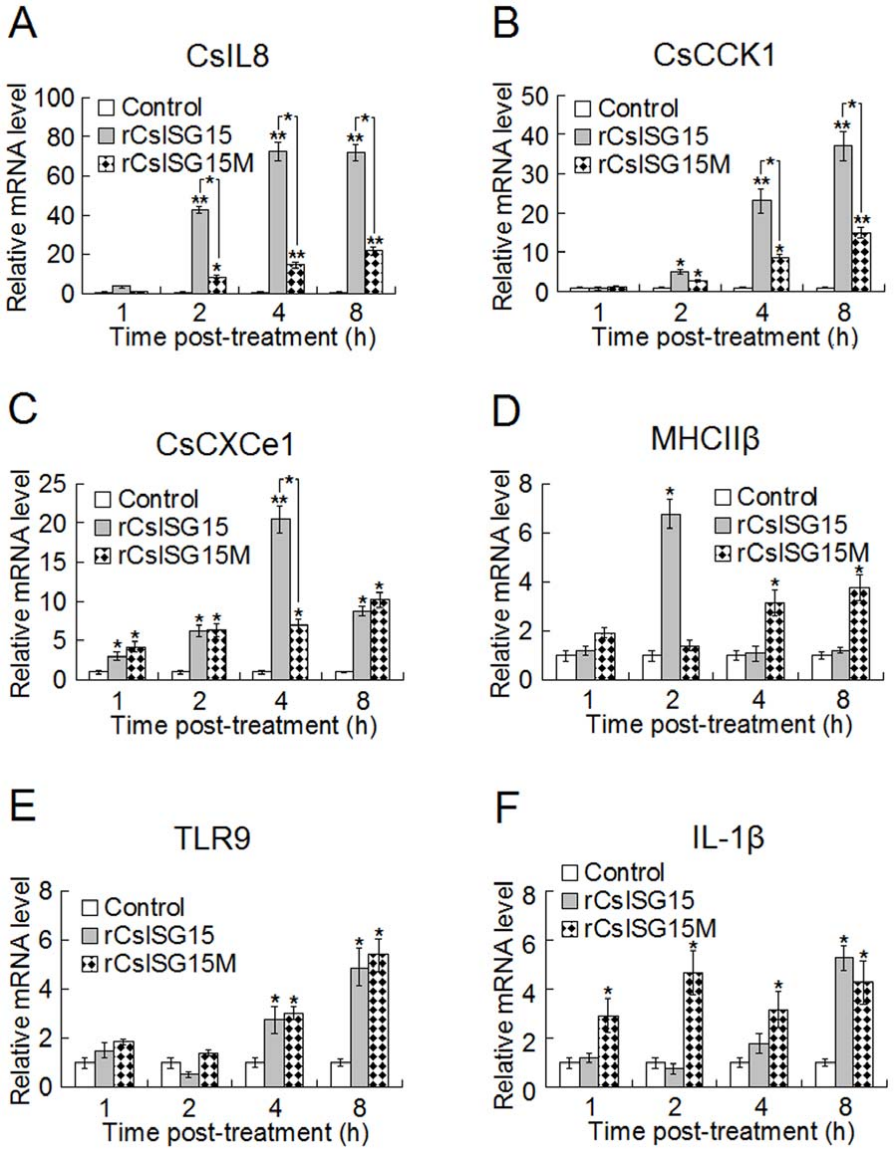
Effect of rCsISG15 and rCsISG15M on the expression of immune genes in lymphocytes. Tongue sole head kidney lymphocytes were treated with or without (control) rCsISG15 or rCsISG15M, and the expression of immune relevant genes was determined by quantitative real time RT-PCR at various time points. The assay was performed three times, and the results are shown as means±SE. ^*^ *P*<0.05; ^**^ *P*<0.01.

To examine the potential effect of rCsISG15 on lymphocyte immune response, HK lymphocytes were treated with or without rCsISG15 or rCsISG15M and then subjected to qRT-PCR analysis of the expression of IL-1β, CsIL8 (a tongue sole homologue of IL-8), TLR9, MHC IIβ, CsCCK1 (a CC chemokine), and CsCXCe1 (a CXC chemokine), which are the major immune relevant genes known in tongue sole. The results showed that treatment with rCsISG15 induced significant inductions of all the examined genes in a time-dependent manner, with drastic inductions (maximum levels of 21–73 fold) occurring in CsIL8, CsCCK1, and CsCXCe1 expression, and moderate inductions (maximum levels of 5–7 fold) occurring in IL-1β, TLR9, and MHC IIβ expression (Fig. 7A). In cells treated with rCsISG15M, the induction patterns of CsIL8, CsCCK1, and CsCXCe1 were similar to those in rCsISG15-treated cells but the induction folds were in general significantly lower than those in rCsISG15-treated cells. No significant difference was observed between the inductions of TLR9 effected by rCsISG15M and rCsISG15. For MHC IIβ and IL-1β, the induction patterns induced by rCsISG15M differed from those induced by rCsISG15. The peak induction of MHC IIβ induced by rCsISG15M was ∼2-fold lower than that induced by rCsISG15, while the peak induction of IL-1β induced by rCsISG15M was comparable to that induced by rCsISG15.

#### (iii) Effect on lymphocyte resistance against viral infection

**Figure 8:**
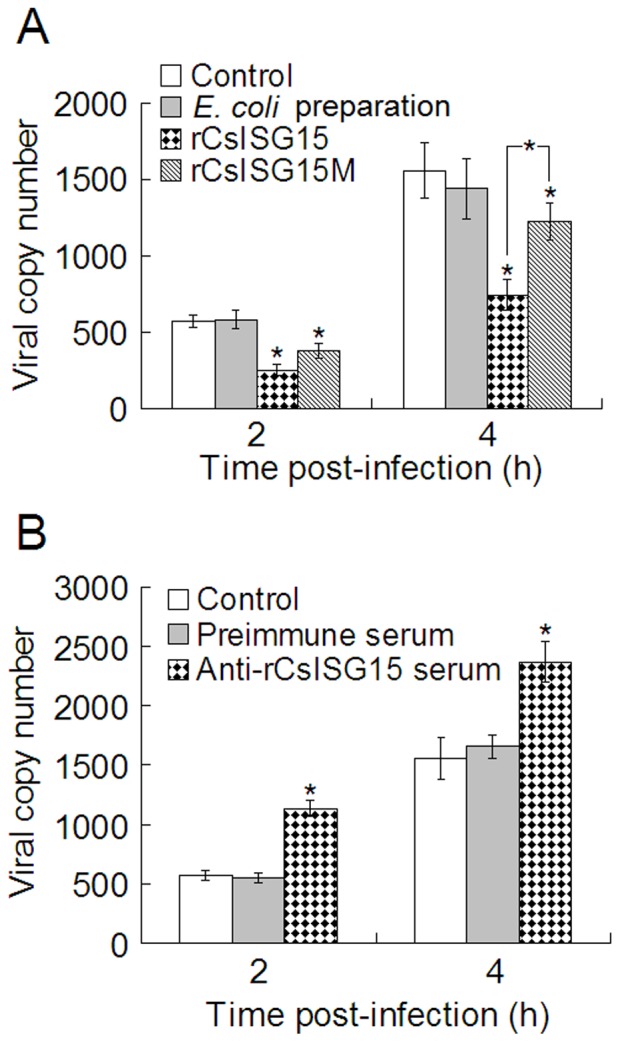
Effect of rCsISG15 and anti-rCsISG15 serum on viral infection. (A) Tongue sole head kidney (HK) lymphocytes were treated with or without (control) rCsISG15, rCsISG15M, or *Escherichia coli* preparation, and then infected with megalocytivirus. (B) Tongue sole HK lymphocytes were pre-treated with or without (control) anti-rCsISG15 serum or preimmune serum and then infected with megalocytivirus. In both panels, viral numbers in the cells were determined at 2 h and 4 h post-infection. Values are shown as means±SE (N = 5). N, the number of repeated experiments. ^*^ *P*<0.05; ^**^ *P*<0.01.

The above results led us to wonder whether the immunoregulatory effect observed with rCsISG15 on lymphocytes would have any effect on cellular resistance against viral infection. To examine this question, HK lymphocytes were treated with or without rCsISG15, rCsISG15M, or *E. coli* preparation before being infected with megalocytivirus. Viral copy numbers in the infected cells were then determined at 2 h and 4 h post-infection. The results showed that at both time points, the viral copies in rCsISG15- and rCsISG15M -treated cells were significantly lower than those in the control cells (Fig. 8A). However, compared to rCsISG15-treated cells, rCsISG15M-treated cells exhibited significantly higher numbers of viral numbers at 4 h post-infection. In contrast, *E. coli* preparation had no effect on viral infection.

### Antibody neutralization of CsISG15 and its effect on viral infection

With the above results, i.e., CsISG15 was released into the culture supernatant of lymphocytes and able to stimulate lymphocyte immune response against viral infection, we wondered whether antibody blocking of the released CsISG15 would have any effect on viral infection. To examine this question, HK lymphocytes were infected with megalocytivirus in the presence or absence of anti-rCsISG15 serum or preimmune serum for 2 h and 4 h. Subsequent determination of intracellular viral number showed that the presence of anti-rCsISG15 serum, but not the presence of preimmune serum, significantly increased intracellular viral load at both examined time points (Fig. 8B).

### Interference with CsISG15 expression and its effect on viral infection

To examine whether interference with CsISG15 expression would have any effect on viral infection, HK lymphocytes were subjected to RNAi assay by being transfected with pRiCsISG15 and pRiNC1, which expressed *CsISG15*-specific siRNA and a nonspecific siRNA respectively. As a control, the cells were also transfected with the backbone vector pRNAT-CMV3.1. To examine the effect of RNAi on CsISG15 expression, the transfectants were infected with megalocytivirus for 4 h, and CsISG15 expression was determined by qRT-PCR and Western blot. qRT-PCR analysis showed that in lymphocytes transfected with pRiCsISG15, *CsISG15* mRNA decreased to 0.9% of that of the control cells, while in lymphocytes transfected with pRiNC1, *CsISG15* mRNA was comparable to that of the control cells. Consistently, Western blot analysis showed that compared to control cells, cells transfected with pRiNC1 produced a comparable amount of extracellular CsISG15, whereas cells transfected with pRiCsISG15 produced no detectable CsISG15 (Figure S4). Taken together, these results indicated that CsISG15 expression was indeed interfered specifically in lymphocytes transfected with pRiCsISG15.

**Figure 9:**
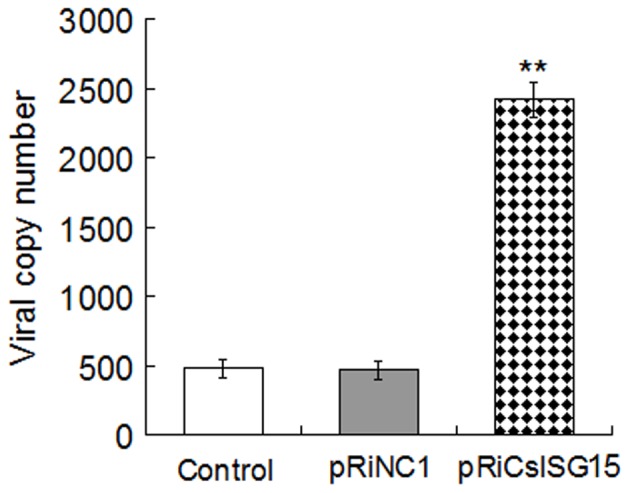
Effect of *CsISG15* RNAi on viral infection. Tongue sole head kidney lymphocytes transfected with pRiCsISG15, pRiNC1, and pRNAT-CMV3.1 (control) were infected with megalocytivirus. Viral numbers in the cells were determined at 4 h post-infection. Data are shown as means±SE (N = 5). N, the number of repeated experiments. Significance between RNAi and the control is indicated with asterisks. ^**^ *P*<0.01.

To determine the effect of RNAi on viral infection, siRNA-treated and untreated lymphocytes were infected with megalocytivirus and analyzed for viral infection. The results showed that the viral number in lymphocytes transfected with pRiCsISG15 was 5.1-fold higher than that in the control cells (Fig. 9). In contrast, the viral number in lymphocytes transfected with pRiNC1 exhibited no apparent difference from that of the control cells.

## Discussion

In this study, we identified CsISG15, an ISG15 homologue from tongue sole, and analyzed its expression and function. Like all known ISG15, CsISG15 possesses two tandem UBL domains and the highly conserved LRGG motif, which categorize CsISG15 as a member of the ISG15 family. In fish, reports have shown that expression of most of the identified ISG15 is enhanced by microbial challenge. For example, viral infection and poly(I:C) treatment activate the expression of the *ISG15* of Atlantic cod [28], [29], [31], Atlantic salmon [27], [32], black rockfish [21], channel catfish [23], crucian carp [26], and Japanese flounder [25], while bacterial challenge activates *ISG15* expression in Atlantic cod [25], goldfish [22], and red drum [30]. In this study, we observed constitutive *CsISG15* expression in a wide range of tissues and drastically enhanced expression of *CsISG15* in kidney and spleen following experimental infection with megalocytivirus and *V. anguillarum*. In both kidney and spleen, the induction patterns caused by viral and bacterial challenges differed, probably due to different activation mechanisms effected by these two pathogens. Consistent with these observations in a live fish model, cellular study showed that megalocytivirus infection induced strong induction of *CsISG15* expression in HK lymphocytes in a time-dependent manner. Like human ISG15, which is known to be secreted into the extracellular milieu, CsISG15 protein was detected in the culture medium of HK lymphocytes following viral challenge. These results indicate that viral challenge stimulates CsISG15 expression at both transcription and translation levels. Since, like all ISG15, CsISG15 lacks an apparent signal sequence, extracellular exportation of CsISG15 is probably accomplished through a non-classical secretion pathway.

It is known that recombinant human ISG15 purified from *E. coli* exhibits cytokine-like activity and is able to induce natural killer cell proliferation and IFN-γ production by T lymphocytes [15]–[18]. In our study, we found that rCsISG15 stimulated the respiratory burst and NO production of HK macrophages and upregulated the expression of immune relevant genes in HK lymphocytes. These results suggest that rCsISG15 possesses immunoregulatory property. Since CsISG15, like some fish ISG15 such as those of *Paralichthys olivaceous*, *Sciaenops ocellatus*, and *Oncorhynchus mykiss*, has a C-terminal extension, which is a seven-residue sequence in CsISG15, beyond the LRGG motif, and this extension was not removed from rCsISG15, it is likely that cleavage of the C-terminal extension, as is observed with mammalian ISG15 during ISGylation, is not necessary for the biological activity of CsISG15 as an extracellular cytokine. Mutational study showed that, in contrast to rCsISG15, the mutant rCsISG15M had no apparent effect on the respiratory burst activity of HK macrophages and, compared to rCsISG15, much reduced though significant effect on NO production. These results suggest that the LRGG motif is required for the optimum activity of CsISG15 as an extracellular cytokine. It is possible that the LRGG motif, besides being participating in conjugate formation with intracellular substrate proteins, has additional functions such as being involved in interactions with cell surface proteins that serve as receptors for extracellular CsISG15.

qRT-PCR analysis showed that rCsISG15 drastically upregulated the expression of CsIL8, CsCCK1, and CsCXCe1, suggesting that rCsISG15 is a strong inducer of both CC and CXC chemokines. In addition to chemokines, rCsISG15 also upregulated the expression of TLR9 and the proinflammatory cytokine IL-1β. These results indicate that rCsISG15 is a stimulator of innate immunity, which may at least in part account for the anti-microbial property of rCsISG15. Compared to rCsISG15, rCsISG15M exhibited, in most cases, significantly lower stimulatory effects on the expression of immune genes, which is in line with the observation that rCsISG15M was impaired in the ability to activate HK macrophages and to block viral infection.

Previous studies have indicated that mammalian ISG15 possess a broad-spectrum antiviral activity against a wide range of DNA and RNA viruses, and in most cases the antiviral activity is dependent on ISGylation of either host or viral proteins [9], [14]. However, a recent study of Chikungunya virus infection in a murine model showed that the antiviral effect of mouse ISG15 is independent on its ability to form conjugates with substrate proteins, which suggests a non-classical role for ISG15 during viral infection [33]. In our study, we found that treatment of HK lymphocytes with rCsISG15 before megalocytivirus infection reduced intracellular viral load, whereas interference with CsISG15 expression drastically increased intracellular viral load, suggesting that rCsISG15 is required for optimum resistance against megalocytivirus infection. Since rCsISG15 induced significant inductions of CsIL8, CsCCK1, CsCXCe1, IL-1β, and TLR9, which are involved in innate immunity against pathogen infection, it is likely that the anti-megalocytivirus effect of rCsISG15 is the result of augmented cellular immune response rather than a direct interaction between rCsISG15 and the viral pathogen. In line with these observations, the presence of rCsISG15 antiserum in the culture medium of HK lymphocytes during megalocytivirus infection increased the intracellular viral load to significant extents, suggesting that interaction between anti-rCsISG15 antibodies and the released CsISG15 most likely blocked the activity of CsISG15, thus resulting in enhanced viral infection.

In conclusion, we showed in this study that CsISG15 is upregulated in expression by microbial pathogens and that, upon viral infection, CsISG15 is released into the extracellular milieu, where the protein probably acts as a cytokine and activates the response of various immune cells, which leads to elevated host defense against viral infection. Hence, it appears that, unlike mammalian ISG15, whose antiviral effects are mediated mostly through intracellular protein modification, CsISG15 exerts its immunoregulatory activity extracellularly as an unconjugated protein. Our results also indicate for the first time that the conserved LRGG motif of a teleost ISG15 is not essential to but required for the optimum activity of ISG15 as an extracellular cytokine, which suggests a role for the LRGG motif other than that in ISGylation. Taken together, these results support the conclusion that CsISG15 functions as an unconjugated extracellular cytokine that promotes antiviral immunity in a LRGG motif-dependent manner.

## Materials and Methods

### Ethics statement

Experiments involving live fish and rats were conducted in accordance with the “Regulations for the Administration of Affairs Concerning Experimental Animals” promulgated by the State Science and Technology Commission of Shandong Province.

### Fish

Tongue sole (*Cynoglossus semilaevis*) were purchased from a commercial fish farm in Shandong Province, China and maintained at 22°C in aerated seawater. Fish were acclimatized in the laboratory for two weeks before experimental manipulation. Before each experiment, fish were randomly sampled for the examination of bacterial recovery from internal organs (blood, liver, kidney, and spleen), and no bacteria were detected from any of the examined fish. Fish were euthanized with tricaine methanesulfonate (Sigma, St. Louis, MO, USA) before tissue collection.

### Cloning of *CsISG15*

A cDNA library of tongue sole head kidney and spleen was constructed as described previously [34]. One thousand and five hundred clones of the library were randomly selected and subjected to DNA sequence analysis; one clone was found to contain the cDNA of *CsISG15* with 5′- and 3′ - untranslated regions (UTRs). The nucleotide sequence of *CsISG15* has been deposited in GenBank database under the accession number JN967757.

### Sequence analysis

The cDNA and amino acid sequences of CsISG15 were analyzed using the BLAST program at the National Center for Biotechnology Information (NCBI) and the Expert Protein Analysis System. Domain search was performed with the simple modular architecture research tool (SMART) version 4.0 and the conserved domain search program of NCBI. The molecular mass and theoretical isoelectric point (pI) were predicted using EditSeq in DNASTAR software package (DNASTAR Inc. Madison, WI, USA). Multiple sequence alignment was performed with the ClustalX program. Signal peptide search was performed with SignalP 3.0.

### Plasmid construction and mutation of CsISG15

To construct pEtCsISG15, which expresses a His-tagged CsISG15, the coding sequence of CsISG15 was amplified by PCR with primers CsISGF1 (5′- GATATCATGGAAATAACCATCACAA -3′; underlined sequence, EcoRV site) and CsISGR1 (5′- GATATCGCCAATGACGGTGT -3′; underlined sequence, EcoRV site); the PCR products were ligated with the T–A cloning vector pBS-T (Tiangen, Beijing, China), and the recombinant plasmid was digested with EcoRV to retrieve the *CsISG15*-containing fragment, which was inserted into pET259 [35] at the EcoRV site. To construct pEtCsISG15M, which expresses a mutant CsISG15, CsISG15M, bearing alanine substitutions at R153 and G154 (so that the conserved LRGG motif was mutated to LAAG), a two-step PCR was carried out as follows: the first PCR was performed with primers CsISGF1 and MR3 (5′ - GCCAATGACGGTGTGTCCTCTGCCTGCTGCCAGACGCAGT -3′); the PCR products were diluted 100×, and the dilution was used as a temperate for the second PCR with primers CsISGF1 and CsISGR1. The PCR products were inserted into pET259 at the EcoRV site as described above, resulting in pEtCsISG15M.

### Purification of recombinant protein and preparation of antiserum

*Escherichia coli* Rosetta (DE3) (Bioteke, Beijing, China) was transformed with pEtCsISG15, pEtCsISG15M, and the empty vector pET259, resulting in transformants DE3/pEtCsISG15, DE3/pEtCsISG15M, and DE3/pET259 respectively. The transformants were cultured in Luria-Bertani broth (LB) medium at 37°C to mid-log phase, and isopropyl-β-D-thiogalactopyranoside was added to the culture to a final concentration of 1 mM. After growth at 18°C for an additional 10 h, recombinant protein was purified using nickel-nitrilotriacetic acid columns (GE Healthcare, USA) as recommended by the manufacturer. Purified proteins and the preparation from DE3/pET259 (named *E. coli* preparation) were dialyzed for 24 h against phosphate-buffered saline (PBS) and concentrated using Amicon Ultra Centrifugal Filter Devices (Millipore, Billerica, MA, USA). The proteins were analyzed by sodium dodecyl sulfate-polyacrylamide gel electrophoresis (SDS-PAGE) and visualized after staining with Coomassie brilliant blue R-250 (Figure S1). The concentrations of the purified proteins were determined using the Bradford method with bovine serum albumin as a standard.

To prepare anti-rCsISG15 antibodies, adult rats (purchased from the Institute for Drug Control, Qingdao, China) were immunized via subcutaneous injection with rCsISG15 mixed in complete Freund's adjuvant. The rats were boosted at 20, 32, and 45 days after the initial immunization. The rats were bled 14 days after the last boost, and sera were obtained from the blood. The titer and specificity of the serum antibodies were determined by enzyme-linked immunosorbent assay and Western immunoblot analysis as described previously [36]. Western immunoblot analysis showed that anti- rCsISG15 serum could bind to rCsISG15 and rCsISG15M but not to purified rCsFerM, a recombinant ferritin of tongue sole [34] (Figure S2 and data not shown).

### CsISG15 expression in fish tissues

#### (i) *CsISG15* expression under normal physiological conditions

Brain, muscle, heart, gut, kidney, spleen, gill, and liver were taken aseptically from five fish (∼12.7 g) and used for total RNA extraction with the RNAprep Tissue Kit (Tiangen, Beijing, China). One microgram of total RNA was used for cDNA synthesis with the Superscript II reverse transcriptase (Invitrogen, Carlsbad, CA, USA). Quantitative real time reverse transcriptase-PCR (qRT-PCR) was carried out in an Eppendorf Mastercycler (Eppendorf, Hamburg, Germany) using the SYBR ExScript qRT-PCR Kit (Takara, Dalian, China) as described previously [37]. PCR efficiency (99.9%) was determined as described previously [38]. Melting curve analysis of amplification products was performed at the end of each PCR to confirm that only one PCR product was amplified and detected. The expression level of *CsISG15* was analyzed using comparative threshold cycle method (2^−ΔΔCT^) with β-actin as a control. The primers used to PCR the β-actin gene were reported previously [39]. The primers used to PCR *CsISG15* were CsISG15RTF1 (5′ –CTACGGTCTGCGTTCTGGC -3′) and CsISG15RTR1 (5′ –CACCCTGCGCTTAAAGTTGG -3′). All PCR were performed in triplicate, and the data are given in terms of mRNA levels relative to that of β-actin and expressed as means plus or minus standard errors of the means (SE).

#### (ii) *CsISG15* expression in response to pathogen infection

The fish bacterial pathogen *Vibrio anguillarum* C312 [37] was cultured in LB medium and resuspended in PBS to 1×10^7^ colony forming units (CFU)/ml. Tongue sole (∼12.7 g) were divided randomly into three groups (N = 30) and injected intraperitoneally with 100 µl PBS containing 10^6^ CFU *V. anguillarum* or 10^4^ copies of the megalocytivirus RBIV-C1 [40] or with 100 µl PBS alone (control). Fish (five for each time point) were sacrificed at 1 h, 4 h, 12 h, 24 h, and 48 h post-infection, and tissues were taken under aseptic conditions. For mRNA expression analysis, total RNA was extracted and used for qRT-PCR as described above. For Western blot analysis, proteins were extracted from 33 mg of tissues with the Sample Grinding Kit (GE Healthcare, New Jersey, USA), and equal volumes of protein preparations were loaded into SDS-PAGE gels. After electrophoresis, the proteins were transferred to a nitrocellulose membrane (Amersham, Cambridge, UK). Immunoblot was performed as described previously [41] using anti-rCsISG15 antibodies or anti-β-actin antibody (Bioss, Beijing, China).

### CsISG15 expression and localization in head kidney (HK) lymphocytes

HK lymphocytes were prepared as described previously [42] and cultured in 96-well tissue culture plates containing L-15 medium (Thermo Scientific HyClone, Beijing, China) supplemented with 10% calf serum. The culture was maintained at 25°C. To examine the effect of viral infection on *CsISG15* expression, megalocytivirus RBIV-C1 was suspended in L-15 to 10^5^ copies/ml. HK lymphocytes were washed and added with fresh L-15 containing or not containing megalocytivirus (10^4^ copies/well). The cells were incubated at 25°C for 1 h, 2 h, 4 h, 8 h, and 16 h respectively and washed 3× with PBS. The cells were then examined for *CsISG15* mRNA expression and extracellular production of CsISG15 protein. For mRNA expression analysis, total RNA was prepared from the cells with Total RNA Kit I of Omega Bio-tek (Norcross, GA, USA) and used for qRT-PCR analysis of *CsISG15* expression as described above. For extracellular protein production analysis, the culture supernatant of 5×10^6^ cells was collected by centrifugation and concentrated with Amicon Ultra Centrifugal Filter Devices (Millipore, Billerica, MA, USA). The proteins were resolved by SDS-PAGE and, after electrophoresis, transferred to a nitrocellulose membrane (Amersham, Cambridge, UK). Immunoblot was performed as described previously [41] using anti-rCsISG15 antibodies or anti-β-actin antibody (Bioss, Beijing, China). To demonstrate that the β-actin antibody used in the study was able to detect β-actin when it was present, cytoplasmic proteins were prepared and blotted with β-actin antibody.

### Effect of rCsISG15 on macrophage activation

#### (i) Preparation of HK macrophages

HK macrophages were prepared based on the method of Chung and Secombes [43]. Briefly, HK was removed from two healthy tongue sole (∼720 g), washed 3× with PBS, and passed through a sterile metal mesh. The cells were resuspended in L-15 medium and placed onto a 34/51% Percoll (Solarbio, Beijing, China) gradient, followed by centrifugation at 400× *g* for 30 min. After centrifugation, the cells at the 34/51% interface were recovered, washed twice and resuspended in L-15 medium containing 10% calf serum, 1% penicillin and streptomycin (Sangon, Shanghai, China), and 20 U/ml heparin (Sigma, St. Louis, MO, USA). The cells were added to 96-well tissue culture plates (∼2×10^5^ cells/well) and incubated at 25°C for 2 h. Non-adherent cells were washed off after the incubation.

#### (ii) Respiratory burst assay

rCsISG15 and rCsISG15M were diluted in L-15 medium to 1000 ng/ml. Macrophages were added with or without rCsISG15, rCsISG15M, or *E. coli* lipopolysaccharide (LPS) (Sigma, St. Louis, MO, USA) in L-15 to a final concentration of 100 ng/ml. After incubation at 25°C for 2 h, respiratory burst activity was determined as reported previously [43]. In brief, 100 µl of 1 mg/ml nitroblue tetrazolium (Sangon, Shanghai, China) in L-15 was added to macrophages. After incubation at 25°C for 2 h, the reaction was stopped by adding 100% methanol. The plate was washed with 70% methanol, and the reduced formazan was solubilized in 100 µl of 2 M KOH and 120 µl of DMSO. The plate was read at 630 nm with a microplate reader.

#### (iii) Nitrite assay

HK macrophages as described above were added with or without rCsISG15 or rCsISG15M in L-15 to a final concentration of 100 ng/ml. After incubation at 25°C for 2 h, nitric oxide (NO) production was determined as described by Tafalla and Novoa [44]. In brief, the supernatants were removed to a separate 96-well plate (50 µl/well); one-hundred microlitres of 1% sulphanilamide (Sigma, St. Louis, MO, USA) in 2.5% phosphoric acid was added to each well, followed by adding 100 µl of 0.1% N-naphthylethylene-diamine (Sigma) in 2.5% phosphoric acid. The plate was read at 540 nm, and the molar concentration of nitrite was determined from standard curves generated using known concentrations of sodium nitrate.

### Effect of rCsISG15 and rCsISG15M on immune gene expression

HK lymphocytes prepared above were added with or without rCsISG15 or rCsISG15M in L-15 to a final concentration of 100 ng/ml. The plates were incubated at 25°C for 1 h, 2 h, 4 h, and 8 h, and total RNA was prepared from the cells with the Total RNA Kit I of Omega Bio-tek (Beijing, China) and used for qRT-PCR analysis of the expression of the genes encoding toll-like receptor 9 (TLR9), interleukin (IL) 1β (IL-1β), CsIL8 (a homologue of IL-8) [39], CsCCK1 (a CC chemokine) [45], CsCXCe1 (a CXC chemokine) [46], and major histocompatibility complex (MHC) class IIβ as described above. These genes were selected because they are the major immune relevant genes that are available in sequence. The primers used to PCR the immune genes have been reported previously [46].

### Effect of rCsISG15 and rCsISG15M on cellular resistance against viral infection

HK lymphocytes prepared above were added with or without rCsISG15 or rCsISG15M in L-15 to a final concentration of 100 ng/ml. As a control, the preparation from DE3/pET259, i.e., *E. coli* preparation, prepared above under the same conditions as rCsISG15 was also added to lymphocytes at the same volume as rCsISG15. The plates were incubated at 25°C for 2 h. After the incubation, the cells were added with L-15 containing or not containing megalocytivirus (10^4^ copies/well). The plates were incubated at 25°C for 2 h and 4 h respectively, followed by washing 3× with PBS. Viral copy number in the infected cells was determined by absolute quantitative real time PCR as reported previously [40].

### Effect of anti-rCsISG15 serum on viral infection

Anti-rCsISG15 serum and preimmune serum were diluted 2000× in L-15. HK lymphocytes prepared above were added with or without 100 µl of the diluted anti-rCsISG15 serum or preimmune serum, followed by adding 100 µl L-15 containing or not containing megalocytivirus (10^4^ copies/well). The plates were incubated at 25°C for 2 h and 4 h respectively. After incubation, the plates were washed 3× with PBS, and intracellular viral copy number was determined by absolute quantitative real time PCR as reported previously [40].

### RNAi and its effect on viral infection

RNAi was performed with the DNA vector-based siRNA technology. For this purpose, the siRNA expression vector pRNAT-CMV3.1 (GenScript, Piscataway, NJ, USA) was used, in which the CMV promoter drives the expression of siRNA that can be inserted into the vector between BamH I and Afl II sites. In our study, the *CsISG15*-specific siRNA (5′- GCTCTGAGCACCATTGAGC -3′) and a nonspecific siRNA (5′- GCTTGATGCTCACGACGAC -3′) were inserted into pRNAT-CMV3.1, resulting in plasmids pRiCsISG15 and pRiNC1 respectively. RNAi was carried out by transient transfection of HK lymphocytes with pRiCsISG15, pRiNC1, and pRNAT-CMV3.1 (backbone vector) respectively using Lipofect (Tiangen, Beijing, China) according to manufacturer's instructions. The transfectants were infected with megalocytivirus for 4 h and then examined for CsISG15 expression by qRT-PCR and immunoblot analysis as described above. To examine the effect of RNAi on viral infection, pRiCsISG15-, pRiNC1-, and pRNAT-CMV3.1-transfected cells in 96-well culture plates were infected with megalocytivirus for 4 h and determined for intracellular viral load as described above. The assay was performed independently for five times.

### Statistical analysis

All statistical analyses were performed with SPSS 15.0 software (SPSS Inc., Chicago, IL, USA). Data were analyzed with analysis of variance (ANOVA), and statistical significance was defined as *P*<0.05.

## Supporting Information

Figure S1 **SDS-PAGE analysis of rCsISG15 and rCsISG15M.** Purified rCsISG15 (lane 2) and rCsISG15M (lane 3) were analyzed by SDS-PAGE and viewed after staining with Coomassie blue. Lane 1, protein marker.
(TIF)

Figure S2 **Western blot analysis of the specificity of anti-rCsISG15 antibodies.** rCsISG15 (lane 2) and rCsFerM (lane 3) were subjected to SDS-PAGE (upper panel) or immunoblot analysis with antiserum against rCsISG15 (lower panel). Lane 1, protein marker.
(TIF)

Figure S3 **Alignment of the amino acid sequences of ISG15 homologues.** Dots denote gaps introduced for maximum matching. Numbers in brackets indicate overall sequence identities between CsISG15 and the compared sequences. The residues that are conserved among all the aligned sequences are in black; the residues that are ≥75% identical among the aligned sequences are in grey. The GenBank accession numbers of the aligned sequences are as follows: *Oplegnathus fasciatus* (BAJ16365), *Epinephelus coioides* (AEG78371), *Paralichthys olivaceus* (BAI48419), *Sebastes schlegelii* (BAG72218), *Anoplopoma fimbria* (ACQ57871), *Channa argus* (ABK63480), *Sciaenops ocellatus* (ADJ57326), *Oncorhynchus mykiss* (NP_001118081), *Salmo salar* (NP_001117112), *Gadus morhua* (ABD60150), *Danio rerio* (NP_001191098), *Homo sapiens* (AAH09507), *Mus musculus* (AAH31424).
(TIF)

Figure S4 **CsISG15 production in lymphocytes subjected to RNAi.** Tongue sole head kidney lymphocytes transfected with pRNAT-CMV3.1 (control), pRiNC1, and pRiCsISG15 (lanes 2, 3, and 4 respectively) were infected with megalocytivirus for 4 h. Extracellular (A and B) and cytoplasmic (C and D) proteins were prepared and subjected to immunoblot with antibodies against rCsISG15 (A and C) or β-actin (B and D). Lane 1, protein marker.
(TIF)
