# Supplementary figures and images for: Cynoglossus semilaevis ISG15: A Secreted Cytokine-Like Protein That Stimulates Antiviral Immune Response in a LRGG Motif-Dependent Manner
Source: PLoS One. 2012 Sep 18;7(9):e44884. doi: 10.1371/journal.pone.0044884 (PMC3445607; doi:10.1371/journal.pone.0044884)

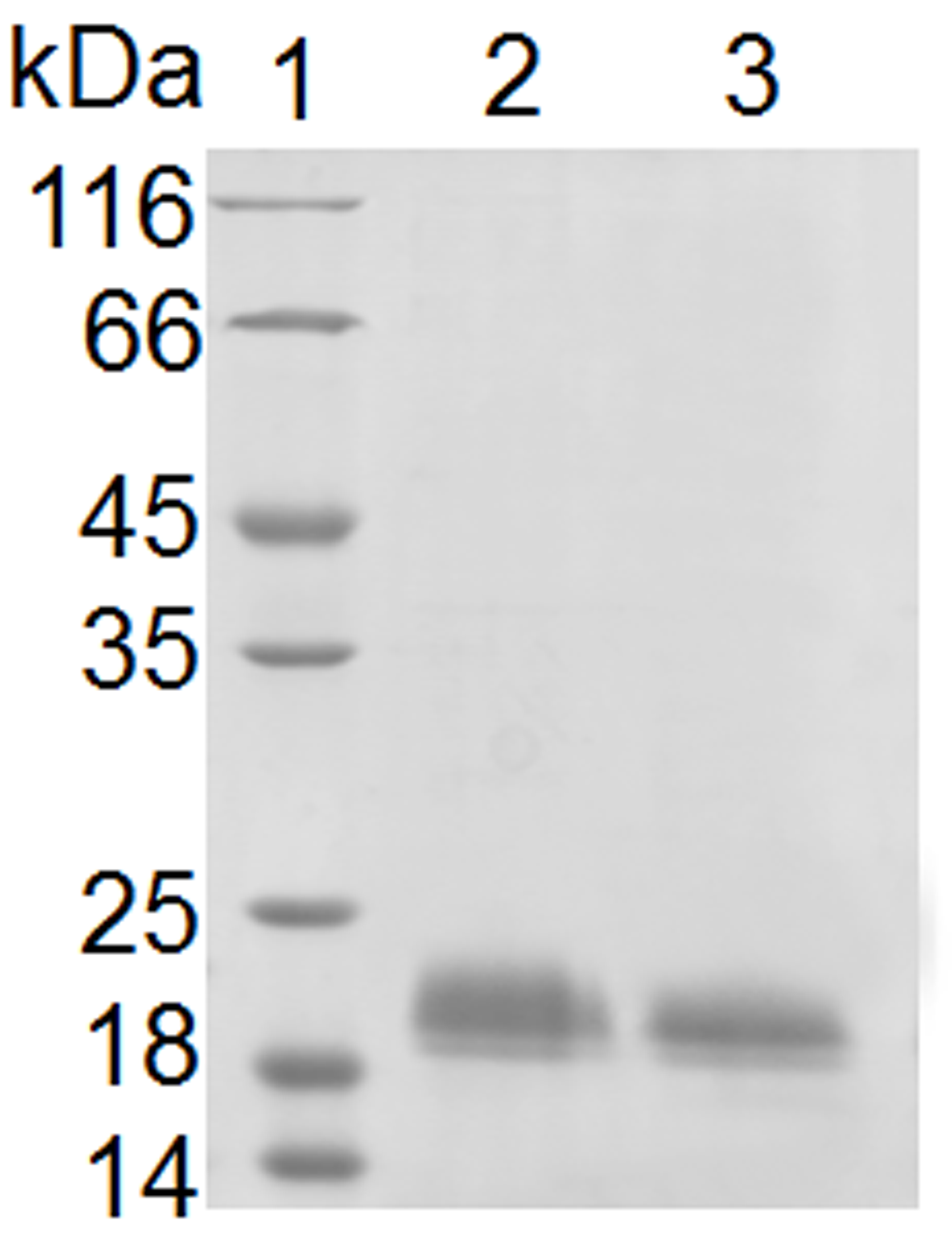

Supplement: Figure S1 — SDS-PAGE analysis of rCsISG15 and rCsISG15M. Purified rCsISG15 (lane 2) and rCsISG15M (lane 3) were analyzed by SDS-PAGE and viewed after staining with Coomassie blue. Lane 1, protein marker. (TIF) [file pone.0044884.s001.tif]

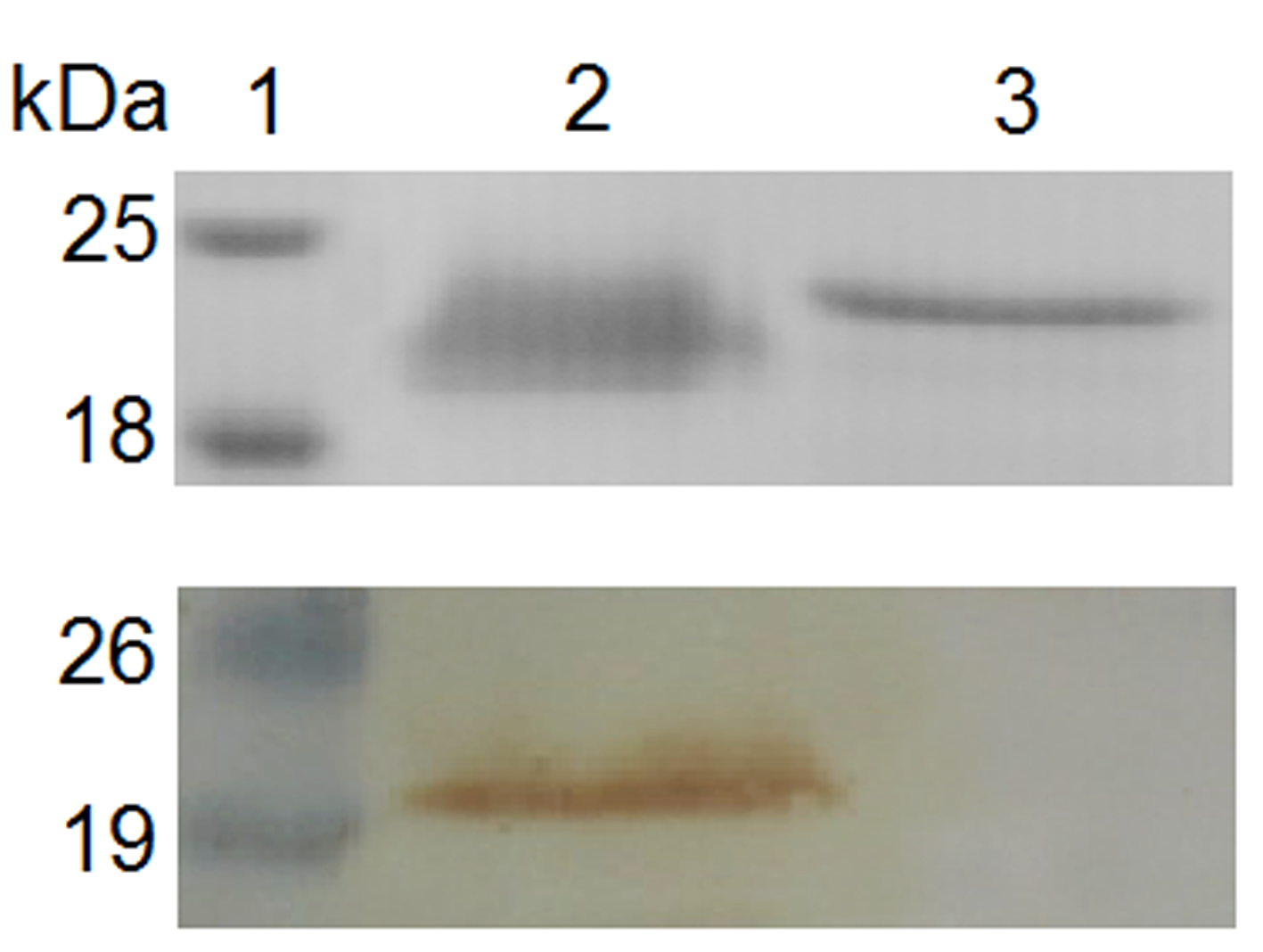

Supplement: Figure S2 — Western blot analysis of the specificity of anti-rCsISG15 antibodies. rCsISG15 (lane 2) and rCsFerM (lane 3) were subjected to SDS-PAGE (upper panel) or immunoblot analysis with antiserum against rCsISG15 (lower panel). Lane 1, protein marker. (TIF) [file pone.0044884.s002.tif]

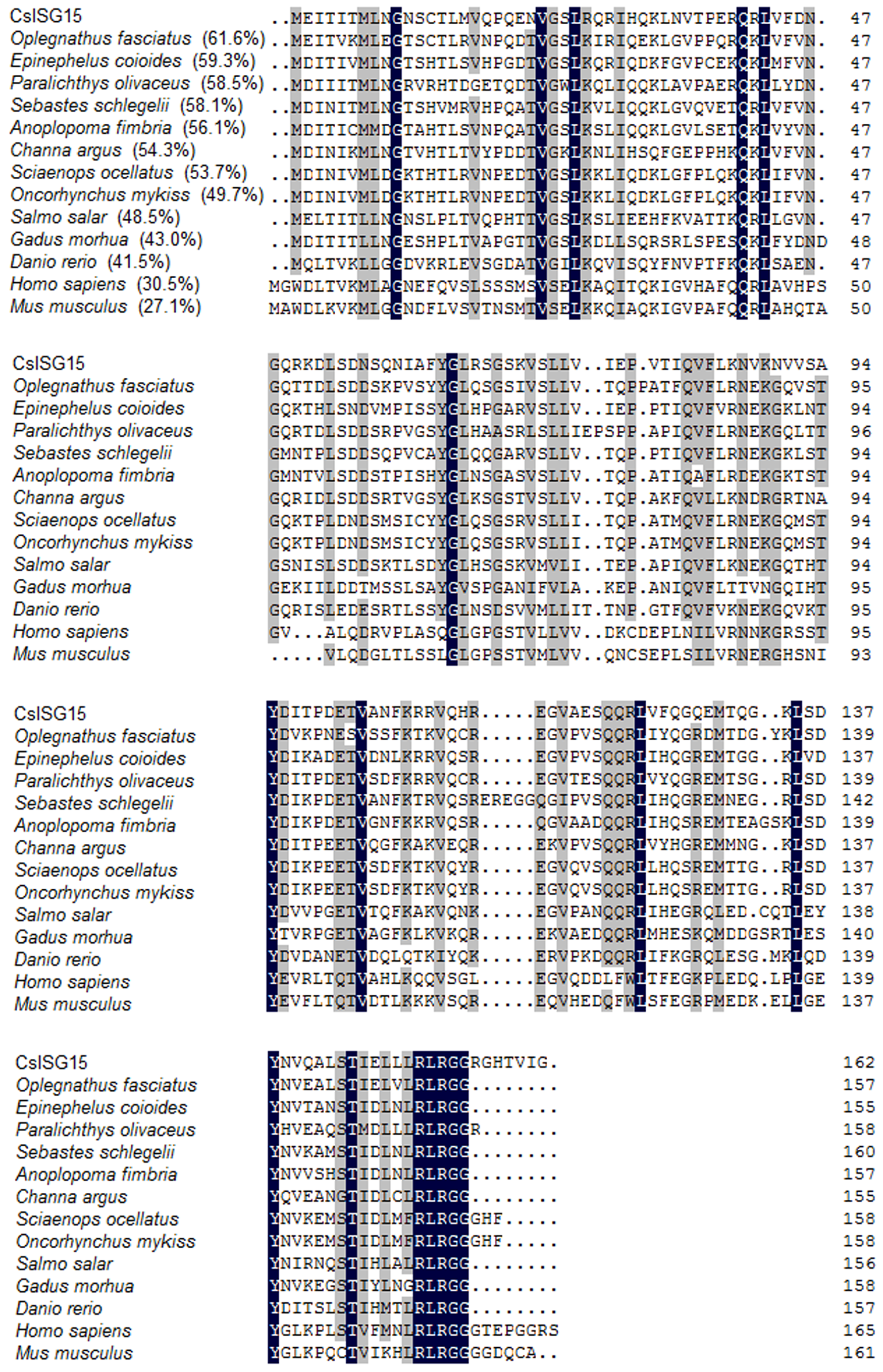

Supplement: Figure S3 — Alignment of the amino acid sequences of ISG15 homologues. Dots denote gaps introduced for maximum matching. Numbers in brackets indicate overall sequence identities between CsISG15 and the compared sequences. The residues that are conserved among all the aligned sequences are in black; the residues that are ≥75% identical among the aligned sequences are in grey. The GenBank accession numbers of the aligned sequences are as follows: Oplegnathus fasciatus (BAJ16365), Epinephelus coioides (AEG78371), Paralichthys olivaceus (BAI48419), Sebastes schlegelii (BAG72218), Anoplopoma fimbria (ACQ57871), Channa argus (ABK63480), Sciaenops ocellatus (ADJ57326), Oncorhynchus mykiss (NP_001118081), Salmo salar (NP_001117112), Gadus morhua (ABD60150), Danio rerio (NP_001191098), Homo sapiens (AAH09507), Mus musculus (AAH31424). (TIF) [file pone.0044884.s003.tif]

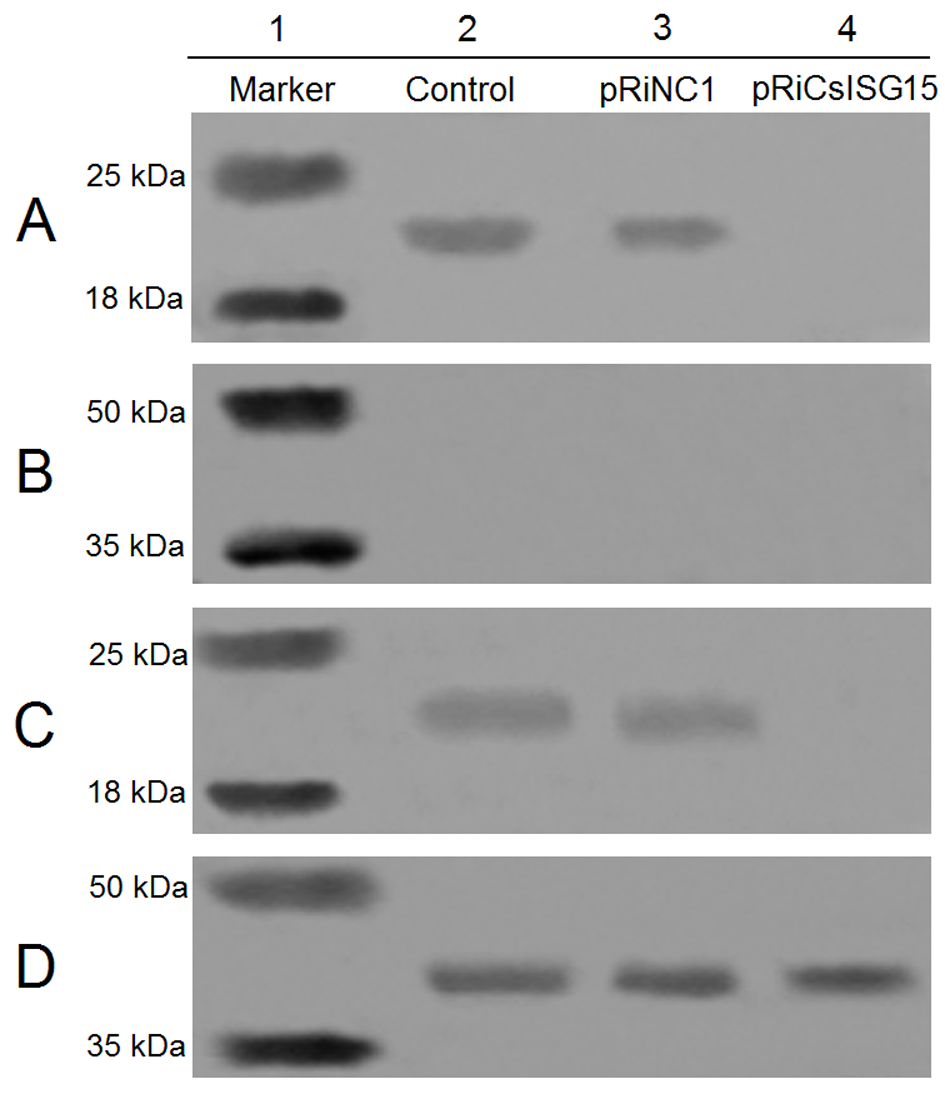

Supplement: Figure S4 — CsISG15 production in lymphocytes subjected to RNAi. Tongue sole head kidney lymphocytes transfected with pRNAT-CMV3.1 (control), pRiNC1, and pRiCsISG15 (lanes 2, 3, and 4 respectively) were infected with megalocytivirus for 4 h. Extracellular (A and B) and cytoplasmic (C and D) proteins were prepared and subjected to immunoblot with antibodies against rCsISG15 (A and C) or β-actin (B and D). Lane 1, protein marker. (TIF) [file pone.0044884.s004.tif]
